# Stroke Rates and Clinical Outcomes After Transcaval Transcatheter Aortic Valve Replacement With and Without Central Embolic Protection: A Single-Center Experience

**DOI:** 10.1016/j.jscai.2026.105375

**Published:** 2026-05-14

**Authors:** Tyler Andrews, Victoria Coombe, Ritik Patel, Georgi Fram, Ahmad Jabri, James Lee, John Dawdy, Bryan Zweig, Tiberio M. Frisoli, Pedro Villablanca, Brian P. O’Neill, Pedro Engel Gonzalez

**Affiliations:** a Division of Cardiovascular Medicine, Henry Ford Hospital, Detroit, Michigan; b Wayne State University School of Medicine, Detroit, Michigan; c Department of Cardiovascular Medicine, William Beaumont University Hospital, Royal Oak, Michigan

**Keywords:** alternative access transcatheter aortic valve replacement, cerebral embolic protection, stroke, transcaval transcatheter aortic valve replacement

## Abstract

**Background:**

Alternate access transcatheter aortic valve replacement (TAVR) is associated with higher stroke rates. However, the use of cerebral embolic protection (CEP) in alternative access TAVR is not well defined. We sought to describe the 30-day stroke incidence and clinical outcomes in patients undergoing transcaval TAVR with and without CEP.

**Methods:**

We performed a single-center retrospective cohort study of 197 patients who underwent transcaval TAVR from 2014 to 2025. CEP, when anatomically feasible, was employed using the SENTINEL device (Boston Scientific) after its commercial availability in 2017. The primary outcome was 30-day stroke. Secondary outcomes included 30-day major adverse cardiac events (MACE-3 [defined as the composite of nonfatal myocardial infarction, stroke, and cardiac death]).

**Results:**

Of 197 patients, 59 received CEP and 138 did not. Baseline characteristics, including prior stroke and transient ischemic attack, were similar between groups. Overall, 30-day stroke was rare (2.54%) and similar between CEP and non-CEP patients (1.69% vs 2.9%, *P* = .68). Numerically, more patients without CEP experienced a stroke. Secondary outcomes, including 30-day mortality, myocardial infarction, and composite MACE-3, were similarly low across groups. A shorter hospital stay was observed with CEP use.

**Conclusions:**

In the largest description of stroke rates in transcaval TAVR with and without CEP, 30-day stroke and MACE-3 events were similar with and without CEP use. A further study is warranted to describe the protective utility of CEP in high-risk TAVR cohorts.

## Central Illustration

**Figure:**
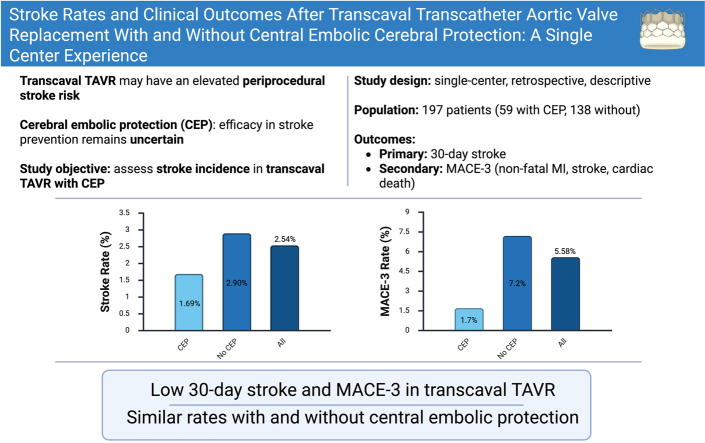

## Introduction

Transcatheter aortic valve replacement (TAVR) has become a standard therapy for patients with symptomatic severe aortic stenosis (AS). However, periprocedural stroke remains a major complication with an incidence of approximately 2% to 4% in contemporary studies,^1^, ^2^, ^3^ contributing to increased morbidity and mortality. The mechanism of stroke in TAVR is complex and not well defined, but some of the risk appears to be intraprocedural with the release of embolic debris during valve deployment. Cerebral embolic protection (CEP) devices have been developed to mitigate this risk by capturing such debris before reaching the cerebral circulation.^4^ However, large randomized controlled trials, including PROTECT TAVR and BHF PROTECT-TAVI, evaluated the use of CEP exclusively in transfemoral TAVR and have not demonstrated a statistically significant reduction in overall stroke rates.^5^^,^^6^

Alternative access is required when there are anatomic or clinical factors precluding the transfemoral approach, and it is often associated with a higher risk of stroke. There is a notable gap in the literature evaluating the efficacy of CEP for alternative access TAVR procedures, particularly the transcaval approach. The transcaval technique has demonstrated promising procedural success rates (>95%)^7^, ^8^, ^9^ and clinical outcomes, including 30-day mortality, 1-year mortality, and major vascular complications, comparable to those of other alternative access approaches such as transaxillary and transcarotid TAVR.^9^^,^^10^ Understanding the stroke risk in this population is critical, and this study aims to address this gap by evaluating the stroke rate in transcaval access TAVR performed with and without CEP.

## Methods

This was a single-center retrospective study of 197 patients who underwent transcaval TAVR from January 1, 2014, to July 1, 2025, at a tertiary valve center. All patients had symptomatic severe AS and underwent comprehensive evaluation by a multidisciplinary cardiac team and were deemed suitable for transcatheter intervention. All patients had prohibitive anatomy for the transfemoral approach and, therefore, underwent TAVR with alternative access. In this cohort, the transcaval TAVR population served as a surrogate for an exceptionally high-risk cohort, characterized by comorbidities associated with an increased risk of periprocedural stroke. This approach aimed to characterize stroke rates across alternate access TAVR in a cohort with a substantial burden of underlying disease, most commonly peripheral vascular disease and extensive aortic and valvular calcification. Although multiple alternate access routes were considered, transcaval access was selected as the most appropriate approach following multidisciplinary evaluation.

At our institution, the SENTINEL CEP device (Boston Scientific) was used in all cases where CEP was employed. This device became commercially available in 2017; therefore, our study includes TAVR procedures performed both before and after its availability. CEP devices were utilized in most cases once they became available and anatomically feasible.

The primary outcome of this study was the incidence of acute clinical stroke within 30-days following TAVR. Routine cerebral imaging was not performed and was only pursued when there was clinical suspicion of stroke or transient ischemic attack (TIA), defined as resolution of stroke-like symptoms within 24-hours. Stroke was confirmed by neurologic evaluation, neuroimaging (radiographic evidence of vessel occlusion or infarct on CT or MRI), and stratified with the National Institutes of Health Stroke Scale (NIHSS)^11^ immediately following clinical diagnosis and performed by neurologists at our center, which is a designated “stroke center of excellence.” Secondary outcomes included 30-day major adverse cardiac events (MACE-3), defined as the composite of nonfatal myocardial infarction (MI),^12^ stroke, and cardiac death. Outcomes were determined through a comprehensive review of electronic medical records and adjudicated retrospectively by the structural heart team to ensure accuracy of postprocedural events. An additional series of descriptive statistics was performed on a high-risk subgroup of patients, defined as per the Society of Thoracic Surgeons (STS) mortality >8% and a heart team risk assessment of high.

Continuous variables are expressed as mean ± SD, and categorical variables are expressed as number (percentage). No inferential statistical tests were performed, and no *P* values were reported. Absolute risks with 95% CIs were calculated for key clinical outcomes.

The methodology of this study was reviewed and approved by the Henry Ford Health Institutional Review Board, ensuring compliance with ethical guidelines and patient confidentiality.

## Results

### Baseline characteristics and procedural data

The study cohort included 197 patients undergoing TAVR, of whom 59 (30.0%) received CEP and 138 (70.0%) did not. The mean age of the overall cohort was 78.1 ± 9.3 years, with CEP patients slightly younger than those without CEP (75.2 ± 9.8 vs 79.4 ± 8.8 years, *P* = .004). Men were more prevalent in the CEP group compared with no CEP (52.5% vs 31.9%, *P* = .006), as presented in Table 1.

**Table 1:** Baseline characteristics and procedural data of patients undergoing TAVR in the overall cohort and stratified by use of CEP.

Characteristics	All (N = 197)	CEP (n = 59)	No CEP (n = 138)	*P* value
Male sex	75 (38.1)	31 (52.5)	44 (31.9)	.006
Age, y	78.1 ± 9.3	75.2 ± 9.8	79.4 ± 8.84	.004
Race				
White	169 (85.8)	54 (91.5)	115 (83.3)	.13
Black	12 (6.1)	3 (5.08)	9 (6.5)	.70
Hispanic or Latino	2 (1.0)	0 (0.00)	2 (1.4)	.35
Other	4 (2.0)	1 (1.69)	3 (2.2)	.83
Unknown	10 (5.1)	1 (1.69)	9 (6.5)	.16
Body mass index, kg/m^2^	27.6 ± 6.5	28.4 ± 6.9	27.3 ± 6.34	.28
Hypertension	175 (88.8)	54 (91.5)	121 (87.7)	.43
Hyperlipidemia	125 (63.5)	44 (74.6)	81 (58.7)	.06
Diabetes	74 (37.6)	22 (37.3)	52 (37.7)	.96
Coronary artery disease	129 (65.5)	42 (71.2)	87 6 (3.0)	.27
Prior myocardial infarction	44 (22.3)	14 (23.7)	30 (21.7)	.76
Congestive heart failure	114 (57.9)	36 (61.0)	78 (56.5)	.56
Atrial fibrillation	74 (37.6)	20 (33.9)	54 (39.1)	.49
Peripheral arterial disease	67 (34.0)	18 (30.5)	49 (35.5)	.50
Carotid	23 (11.7)	6 (10.2)	17 (12.3)	.67
Estimated glomerular filtration rate, mL/min/1.73 m^2^	57.3 ± 21.7	62.0 ± 23.9	55.4 ± 20.6	.06
Prior stroke	18 (9.1)	6 (10.2)	12 (8.7)	.74
Prior transient ischemic attack	6 (3.0)	3 (5.08)	3 (2.2)	.28
Lung disease	54 (27.4)	18 (30.5)	36 (26.1)	.52
Chronic hypoxic respiratory failure	16 (8.1)	3 (5.08)	13 (9.4)	.31
Liver disease	4 (2.0)	1 (1.69)	3 (2.2)	.83
Anticoagulation	26 (13.2)	6 (10.2)	20 (14.5)	.41
Antiplatelet	76 (38.6)	21 (35.6)	55 (39.9)	.57
STS mortality, %	6.67 ± 4.8	5.8 ± 4.4	7.06 ± 4.90	.10
STS morbidity/mortality, %	27.8 ± 12.6	25.3 ± 16.1	28.9 ± 10.6	.07
NYHA functional classification	2.99 ± 0.8	2.9 ± 0.8	3.04 ± 0.77	.18
LVEF, %	55.6 ± 14.7	55.0 ± 13.7	55.9 ± 15.2	.71
Aortic mean gradient, mm Hg	32.5 ± 13.9	30.6 ± 10.9	33.3 ± 15.0	.25
Functional bicuspid aortic valve	19 (9.64)	11 (18.6)	8 (5.8)	.01
Valve-in-valve procedure	2 (1.0)	2 (3.39)	0 (0)	.03
Balloon expandable implant	153 (77.7)	48 (81.4)	105 (76.1)	.42
Self-expanding implant	31 (15.7)	8 (13.6)	23 (16.7)	.58
Mechanical expandable implant	13 (6.6)	3 (5.08)	10 (7.2)	.58
Procedural predilation	37 (18.8)	3 (5.08)	34 (24.6)	.00
Procedural postdilation	12 (6.1)	3 (5.08)	9 (6.5)	.70
Second valve	0 (0)	0 (0)	0 (0)	–
Concomitant PCI	7 (3.6)	5 (8.47)	2 (1.4)	.01
Procedural success	174 (88.3)	50 (84.8)	124 (89.9)	.31

Values are n (%) or mean ± SD. Comparisons between the CEP and no CEP groups were performed using independent 2-tailed *t-*tests for continuous variables and χ^2^ or Fisher exact tests for categorical variables, as appropriate.

BMI, body mass index; CEP, cerebral embolic protection; LVEF, left ventricular ejection fraction; NYHA, New York Heart Association; PCI, percutaneous coronary intervention; STS, Society of Thoracic Surgeons; TAVR, transcatheter aortic valve replacement.

The racial distribution was predominantly White (85.8%). Body mass index and the prevalence of comorbidities, including hypertension, diabetes, coronary artery disease, prior MI, congestive heart failure, atrial fibrillation, peripheral arterial disease, lung or liver disease, and use of anticoagulation or antiplatelet therapy, were similar between the CEP and no CEP groups. Prior cerebrovascular events were similar between groups, with prior stroke occurring in 9.1% of patients overall (10.2% in CEP vs 8.7% in no CEP, *P* = .74) and prior TIA in 3.0% (5.1% in CEP vs 2.2% in no CEP, *P* = .28), Table 1.

The STS predicted risk scores were similar between the CEP and no CEP groups (STS mortality: 5.8 ± 4.4 vs 7.1 ± 4.9, *P* = 0.10; STS morbidity/mortality: 25.3 ± 16.1 vs 28.9 ± 10.6, *P* = .07), as well as heart team risk assessment (moderate risk 6.78% vs 5.80%, high risk 93.9% vs 94.2%), respectively. Echocardiographic parameters, including left ventricular ejection fraction and aortic mean gradient, were also comparable (Table 1).

Notable procedural differences included a higher prevalence of bicuspid valves (18.6% vs 5.8%, *P* = .01), valve-in-valve procedures (3.4% vs 0%, *P* = .03), and concomitant percutaneous coronary intervention (8.5% vs 1.4%, *P* = .01) in the CEP group. Procedural predilation was more common in the no CEP group (24.6% v. 5.1%, *P* < .001). There was a similar distribution of valve type (balloon, self-, or mechanical expandable), postdilation, second valve implantation, or overall procedural success (Table 1).

### Thirty-day stroke outcomes

The primary outcome of acute stroke within 30-days occurred in 5 patients (2.5%) in the overall cohort. Among patients who received CEP, stroke occurred in 1 patient (1.7%; 95% CI, 0.04%-9.09%), compared with 4 patients (2.9%; 95% CI, 0.80%-7.26%) among those without CEP, as shown in the Central Illustration. The absolute risk difference was –1.2% (95% CI, –5.74% to 6.31%, *P* = 1.00). All strokes were ischemic in nature, with no hemorrhagic strokes or TIA reported. The mean NIHSS score among patients experiencing stroke was 4.2 ± 3.0, with scores of 5.0 in the CEP group and 4.0 ± 3.5 in the no CEP group. All strokes were confirmed by neuroimaging. These findings indicate a low overall incidence of 30-day cerebrovascular events following TAVR and a similar rate between patients who received CEP and those who did not. Stroke events and associated data are present in Table 2.

**Central Illustration:**
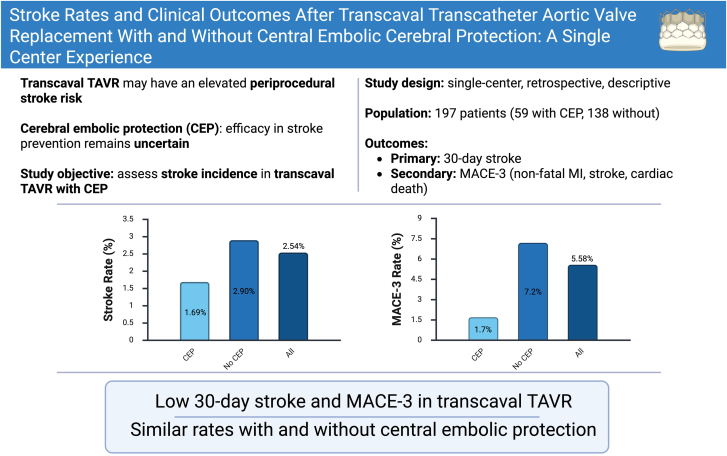
**In asingle-centerstudy of 197 patients undergoing transcaval transcatheter aortic valve replacement (TAVR), there were similar stroke rates and major adverse cardiac events (stroke, nonfatal myocardial infarction, or cardiac mortality) at 30 days with and without cerebral embolic protection (CEP).** MACE-3, major adverse cardiac events (defined as the composite of nonfatal myocardial infarction, stroke, and cardiac death).

**Table 2:** Thirty-day cerebrovascular events following TAVR in the overall cohort and stratified by use of CEP.

	All (N = 197)	CEP (n = 59)	No CEP (n = 138)	*P* value
Any stroke	5 (2.54)	1 (1.69)	4 (2.90)	>.99
Ischemic stroke	5 (100)	1 (20)	4 (80)	
Hemorrhagic stroke	0 (0)	0 (0)	0 (0)	
TIA	0 (0)	0 (0)	0 (0)	
NIHSS score	4.2 ± 3.0	5 ± 0.0	4 ± 3.46	
Major stroke	2 (40)	1 (100)	1 (25)	
Imaging confirmed	5 (100)	1 (100)	4 (100)	

Values are n (%) or mean ± SD.

CEP, cerebral embolic protection; NIHSS, National Institutes of Health Stroke Scale; TIA, transient ischemic attack; TAVR, transcatheter aortic valve replacement.

In a subgroup analysis restricted to high-risk patients, 30-day acute stroke occurred in 2 patients (3.33%), as shown in Table 3. Of these events, 1 stroke (6.25%; 95% CI, 0.2%-30.2%) occurred in the CEP group and 1 (2.27%; 95% CI, 0.1%-12.2%) in the non-CEP group. Both events were ischemic in nature, with a mean NIHSS score of 4.0 ± 1.41.

**Table 3:** Thirty-day cerebrovascular events following TAVR in the high-risk cohort and stratified by use of CEP.

	All (N = 60)	CEP (n = 16)	No CEP (n = 44)
Any stroke	2 (3.33)	1 (6.25)	1 (2.27)
Ischemic stroke	2 (100)	1 (100)	4 (100)
Hemorrhagic stroke	0 (0)	0 (0)	0 (0)
TIA	0 (0)	0 (0)	0 (0)
NIHSS score	4.0 ± 1.41	5.0 ± 0.0	3.0 ± 0.00
Imaging confirmed	2 (100)	1 (100)	1 (100)

Values are n (%) or mean ± SD.

CEP, cerebral embolic protection; NIHSS, National Institutes of Health Stroke Scale; TIA, transient ischemic attack; TAVR, transcatheter aortic valve replacement.

### Secondary outcomes and 30-day MACE-3

**Figure 1:**
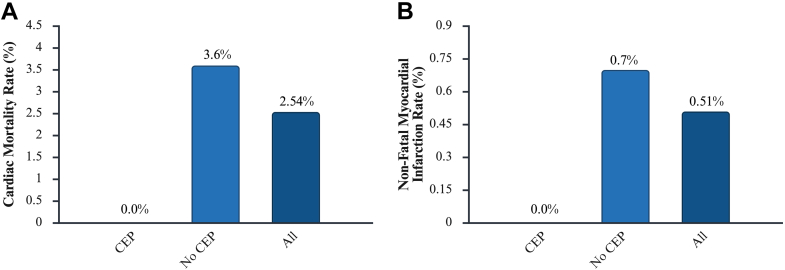
**Thirty-day cardiac mortality and nonfatal myocardial infarction rate.** (**A**) Thirty-day cardiac mortality and (**B**) 30-day nonfatal myocardial infarction following transcaval transcatheter aortic valve replacement, stratified by cerebral embolic protection (CEP) use. The data represent the percentage of patients experiencing events within 30-days of the procedure.

Secondary outcomes, including 30-day MACE-3 events, were low overall. At 30-days, cardiac mortality occurred in 5 patients (2.5%), all in the no CEP group (Figure 1A). Stroke occurred in 5 patients (2.5%), nonfatal MI in 1 patient (0.5%) (Figure 1B), and the composite 30-day MACE-3 occurred in 11 patients (5.6%), as presented in the Central Illustration. MACE-3 events occurred in 1 (1.7%) (95% CI, 0.04%-8.9%) in the CEP group and 10 (7.25%) (95% CI, 3.5%-12.9%) in the no CEP group. This corresponded to a relative risk of 0.23 (95% CI, 0.03-1.78) and an absolute risk difference of –5.6% (95% CI, –12.2% to 2.8%; *P* = .18), as presented in Table 4.^13^

**Table 4:** Clinical outcomes and 30-day MACE-3 following TAVR in the overall cohort and stratified by CEP use.

	All (N = 197)	CEP (n = 59)	No CEP (n = 138)	*P* value
In-hospital mortality	3 (1.52)	0 (0)	3 (2.18)	
Major vascular complication	3 (1.52)	2 (3.4)	1 (0.7)	
Length of stay	5.39 ± 5.7	3.84 ± 3.9	6.06 ± 5.9	
30-day MACE-3	11 (5.58)	1 (1.7)	10 (7.2)	*P* = .18
30-day mortality	5 (2.54)	0 (0)	5 (3.6)	
30-day stroke	5 (2.54)	1 (1.7)	4 (2.9)	
30-day nonfatal myocardial infarction	1 (0.51)	0 (0)	1 (0.7)	

Major vascular complication, defined by VARC-3 criteria.^13^ Values are n (%) or mean ± SD..

CEP, cerebral embolic protection; MACE-3, major adverse cardiac events (defined as the composite of nonfatal myocardial infarction, stroke, and cardiac death); TAVR, transcatheter aortic valve replacement.

In-hospital mortality occurred in 3 patients (1.52%), all in the no CEP group, none of which were attributed to major stroke. Major vascular complications occurred in 3 patients (1.5%), with 2 events in the CEP group and 1 in the no CEP group. The mean length of hospital stay was shorter in the CEP group compared with no CEP (3.8 ± 3.9 vs 6.1 ± 5.9 days), as presented in Table 4.

In a high-risk subgroup, the absolute 30-day stroke risk was 6.25% (95% CI, 0.2%-30.2%) in the CEP group and 2.27% (95% CI, 0.1%-12.0%) in the non-CEP group (Table 5). This corresponded to a relative risk of 2.75 (95% CI, 0.18-42.0) and an absolute risk difference of 4.0% (95% CI, –10.5% to 18.5%). Thirty-day MACE-3 occurred in 6.25% (95% CI, 0.2%-30.2%) of patients receiving CEP and 6.82% (95% CI, 1.4%-18.7%) of those without CEP. This corresponded to a relative risk of 0.92 (95% CI, 0.10-8.6) and an absolute risk difference of –0.6% (95% CI, –15.3% to 14.2%).

**Table 5:** Clinical outcomes and 30-day MACE-3 following TAVR in the high-risk cohort and stratified by CEP use.

	All (N = 60)	CEP (n = 16)	No CEP (n = 44)
In-hospital mortality	2 (3.33)	0 (0)	2 (4.54)
Major vascular complication	1 (1.67)	1 (6.25)	0 (0)
Length of stay	7.19 ± 6.8	4.25 ± 4.1	8.28 ± 7.4
30-day MACE-3	4 (6.67)	1 (6.25)	3 (6.82)
30-day mortality	2 (3.33)	0 (0)	2 (4.54)
30-day stroke	2 (3.33)	1 (6.25)	1 (2.27)
30-day nonfatal myocardial infarction	0 (0)	0 (0)	0 (0)

Values are n (%) or mean ± SD.

CEP, cerebral embolic protection; MACE-3, major adverse cardiac events (defined as the composite of nonfatal myocardial infarction, stroke, and cardiac death); TAVR, transcatheter aortic valve replacement.

## Discussion

This retrospective study at a tertiary valve referral center found similar 30-day stroke rates between transcaval TAVR performed with and without the use of CEP. To our knowledge, this is the largest study describing stroke rates with and without the use of CEP devices in transcaval TAVR, and the largest experience with transcaval TAVR from a single center.

Overall, there is concern that alternative access TAVR is associated with a higher risk of periprocedural stroke.^8^^,^^10^^,^^14^ Frequently, patients who have prohibitive peripheral arterial disease for femoral cannulation have extensive disease, including aortic calcifications, which may increase the overall stroke risk.^15^ One would theorize that use of embolic protection in this high-risk population may have increased utility for stroke reduction, relative to the average-risk patient with AS undergoing standard femoral access. The current literature evaluating CEP during TAVR has been heterogeneous and largely limited to cohorts undergoing standard transfemoral access. Collectively, they have demonstrated numerically lower, but not statistically significant, stroke rates with CEP use. Two large retrospective analyses using nationwide databases show slightly lower stroke rates with CEP vs no CEP in mostly transfemoral TAVR. Notably, CEP was associated with a small, near-significant reduction in stroke associated with death or discharge to nonhome location but was not associated with risk reduction in nondisabling stroke.^16^^,^^17^ Similarly, the PROTECTED TAVR trial reported rates of 2.3% with CEP compared with 2.9% without CEP, also in an all-femoral cohort.^5^ More recently, the BHF PROTECT-TAVI trial found stroke rates of 2.1% with CEP versus 2.2% without, in a cohort of mostly transfemoral TAVR.^6^ Collectively, these studies demonstrate a consistent trend toward reduced stroke rates, especially disabling stroke, in the CEP treatment arm, but none reached statistical significance. However, the lack of statistical significance likely cannot be extrapolated to higher-risk populations, such as in alternative access TAVR.

The overall stroke rate among our cohort was less than that previously described in smaller transcaval cohorts and comparable to that in standard transfemoral access populations. In our study, 2.5% of the 197 patients undergoing transcaval TAVR experienced stroke within 30-days of the procedure. This overall stroke rate is comparable to the transfemoral stroke and/or TIA rate in-hospital in standard transfemoral access,^10^ and periprocedure stroke rate within 30-days, ranging from 1.8% to 2.6%.^5^^,^^17^^,^^18^ Although in studies without routine cerebral imaging, underreporting is always a concern, there is evidence that at centers like ours, with Joint Hospital Commission stroke designation status in Michigan, the stroke rate is significantly higher than in its counterparts. This can be attributed to systems in place that rapidly identify and treat stroke.

Procedural success was observed in 88.3% of patients in this cohort, as defined by VARC-3 technical success criteria.^13^ This is generally consistent with procedural success rates reported in contemporary standard access TAVR literature.^19^^,^^20^ Major vascular complications, most commonly access-site complications and bleeding, are the primary reasons for not meeting technical success criteria.^21^ Our study found comparable stroke incidence with and without the use of CEP devices during transcaval TAVR, and numerically similar stroke rates reported in contemporary standard access CEP literature. It is noteworthy that, numerically, there was only 1 stroke in the CEP group versus 4 in the no CEP group. Although a large analysis of the Nationwide Readmissions Database did not show a significant difference in stroke rate with CEP use, they did report a significant reduction in major strokes (ie, stroke leading to death or discharge to other hospital/nursing facility), 1.2% with CEP vs 1.8% without CEP.^17^ The incidence of major stroke in our study was exceedingly low, occurring only twice in the entire cohort, once among patients treated with CEP and once among those without, thereby limiting the ability to further describe this finding.

Lastly, the similarity in 30-day MACE in our study is consistent with the lack of difference observed in CEP meta-analyses.^22^ However, we did observe a shorter hospital length of stay among patients in the CEP cohort compared with those without CEP use. This trend has been previously reported in cohorts undergoing balloon aortic valvuloplasty with CEP,^23^ suggesting a possible association between embolic protection and favorable postprocedural recovery metrics. The underlying mechanism for this observation remains unclear. One theoretical explanation in our study population is that patients receiving CEP were, on average, younger than those in the no CEP cohort, which may have contributed to faster recovery and earlier discharge. However, this explanation does not fully account for similar findings reported in balloon aortic valvuloplasty cohorts, where age differences were not observed.^23^ Alternatively, expansion of TAVR practice to intermediate risk patients may facilitate earlier mobilization and discharge, though this hypothesis remains speculative. Overall, the mechanism underlying reduced hospital length of stay among CEP cohorts remains uncertain and warrants further investigation.

Overall, evaluating specific patient characteristics may confer greater benefit from CEP use in TAVR and may have a substantial clinical impact by reducing stroke and improving patient morbidity. By identifying high-risk patients and/or characteristics, we can move toward more targeted, effective strategies that maximize the protective potential of CEP and improve outcomes for all TAVR recipients. Although this report describes observed rates in a unique, high-risk TAVR population, comprehensive hypothesis testing on the use of CEP in high-risk groups should be considered to determine utility in this subgroup.

### Limitations

This study has several important limitations that should be considered when interpreting the observed cardiovascular event rates. First, the inclusion of patients treated between 2014 and 2025 introduces potential time-related bias. This period spans changes in the commercial availability of CEP devices, shifts in embolic protection strategies following U.S. Food and Drug Administration approval, and evolving practice patterns influenced by more recent evidence, including the PROTECTED TAVR trial published in 2023. As a result, the routine use of CEP declined at our institution over time, contributing to variability in device utilization and potentially inconsistent practice patterns across the study cohort.

Second, although the transcaval TAVR population was intentionally used as a surrogate for exceptionally high-risk patients, the findings are specific to transcaval access and should be interpreted with caution when extrapolating to standard transfemoral or other alternative access routes. Selection bias may also have occurred, as anatomic constraints may have limited the feasibility of CEP deployment in certain patients, thereby precluding uniform utilization across the cohort.

Additionally, this cohort included procedures performed by multiple operators, introducing potential variability related to individual operator practices. Other procedural factors, such as the use of predilation or postdilation, were not standardized and may have further influenced outcomes.

Finally, given the descriptive nature of this study, the cardiovascular event rates reported are not intended for direct comparative inference but rather to provide insight into outcomes within an alternative access TAVR population that has been relatively underdescribed in the literature. As a retrospective study, this analysis is inherently subject to limitations, including the potential for incomplete event capture and missing outcomes despite routine clinical follow-up. Future studies incorporating hypothesis-driven analyses across other alternative access routes may help clarify whether selected high-risk AS populations derive benefit from CEP that has not been consistently observed in standard access TAVR cohorts.

## Conclusion

In this study, representing the largest single-center description of stroke rates in transcaval TAVR, 30-day stroke was rare and occurred at similar rates between patients with and without CEP. Numerically, more patients in the without CEP group experienced a stroke. Secondary outcomes, including MACE-3, were similarly low across groups. Larger, hypothesis-testing analyses are needed to identify the utility of CEP devices in high-risk subgroups and further recognize predictors to individualize care and optimize neurologic outcomes in alternative access TAVR.

## CRediT authorship contribution statement

**Tyler Andrews:** Conceptualization, Data curation, Formal analysis, Investigation, Methodology, Writing – original draft, Writing – review & editing. **Victoria Coombe:** Writing – original draft, Writing – review & editing. **Ritik Patel:** Writing – original draft, Writing – review & editing. **Georgi Fram:** Conceptualization, Methodology, Investigation. **Ahmad Jabri:** Conceptualization, Investigation, Methodology. **James Lee:** Conceptualization, Investigation, Methodology. **John Dawdy:** Conceptualization, Investigation, Methodology. **Bryan Zweig:** Conceptualization, Investigation, Methodology. **Tiberio M. Frisoli:** Conceptualization, Investigation, Methodology. **Pedro Villablanca:** Conceptualization, Investigation, Methodology. **Brian P. O’Neill:** Conceptualization, Investigation, Methodology. **Pedro Engel Gonzalez:** Conceptualization, Investigation, Methodology, Writing – original draft, Writing – review & editing.
